# Vascular endothelial growth factor A as predictive marker for mTOR inhibition in relapsing high-grade serous ovarian cancer

**DOI:** 10.1186/s12918-016-0278-z

**Published:** 2016-04-18

**Authors:** Peter Andorfer, Alexander Heuwieser, Andreas Heinzel, Arno Lukas, Bernd Mayer, Paul Perco

**Affiliations:** emergentec biodevelopment GmbH, Gersthofer Strasse 29-31, 1180 Vienna, Austria

**Keywords:** Ovarian cancer, Disease modeling, Network biology, Drug resistance, Data integration, Predictive biomarker

## Abstract

**Background:**

Development of resistance against first line drug therapy including cisplatin and paclitaxel in high-grade serous ovarian cancer (HGSOC) presents a major challenge. Identifying drug candidates breaking resistance, ideally combined with predictive biomarkers allowing precision use are needed for prolonging progression free survival of ovarian cancer patients.

Modeling of molecular processes driving drug resistance in tumor tissue further combined with mechanism of action of drugs provides a strategy for identification of candidate drugs and associated predictive biomarkers.

**Results:**

Consolidation of transcriptomics profiles and biomedical literature mining results provides 1242 proteins linked with ovarian cancer drug resistance. Integrating this set on a protein interaction network followed by graph segmentation results in a molecular process model representation of drug resistant HGSOC embedding 409 proteins in 24 molecular processes. Utilizing independent transcriptomics profiles with follow-up data on progression free survival allows deriving molecular biomarker-based classifiers for predicting recurrence under first line therapy. Biomarkers of specific relevance are identified in a molecular process encapsulating TGF-beta, mTOR, Jak-STAT and Neurotrophin signaling. Mechanism of action molecular model representations of cisplatin and paclitaxel embed the very same signaling components, and specifically proteins afflicted with the activation status of the mTOR pathway become evident, including VEGFA. Analyzing mechanism of action interference of the mTOR inhibitor sirolimus shows specific impact on the drug resistance signature imposed by cisplatin and paclitaxel, further holding evidence for a synthetic lethal interaction to paclitaxel mechanism of action involving cyclin D1.

**Conclusions:**

Stratifying drug resistant high grade serous ovarian cancer via VEGFA, and specifically treating with mTOR inhibitors in case of activation of the pathway may allow adding precision for overcoming resistance to first line therapy.

## Background

Development of drug resistance represents a major challenge in cancer therapy. In case of high-grade serous ovarian cancer (HGSOC), accounting for 60–80 % of epithelial ovarian carcinoma, initial sensitivity to standard platinum-based therapy is around 80 %. However, relapse rates in more advanced stages reach up to 70 %, with a first appearance varying from a few months to more than 5 years [1, 2]. In clinical practice a combination with a second drug, mainly taxanes is administered [3]. The benefit in terms of progression free survival of this combination is controversially discussed, but at least toxicity is reduced compared to monotherapy [4]. Further clinical trials have been conducted utilizing drugs for specifically targeting platinum-resistant tumors, but overall response rates were found in a low range of 5–20 % [2].

Several molecular mechanisms leading to drug resistance, being generic across drug classes or specific for a selected drug are described [5]. A prominent factor is increased drug efflux via ABC transporter expression [6]. Other mechanisms limiting drug effect involve modifying the drug or its targets. Examples include formation of glutathione-drug conjugates or expression of tubulin isotypes limiting efficiency of microtubule targeting drugs [7, 8]. Altered downstream effects by e.g. up-regulation of DNA repair mechanisms or anti-apoptotic pathways are a further class of events seen in drug resistance [9]. Deregulation of entire signaling pathways like PI3K-Akt/mTOR are reported as being involved in resistance to antineoplastic agents in a number of tumors [10]. On such background of multiple molecular components individually or in combination contributing to drug resistance, identification of resistance hub functionality on a molecular process level promises optimal targets for halting multiple drug resistance paths. Such strategy demands a systematic integration of the individual resistance contributors into a molecular mechanistic representation of a drug resistant clinical phenotype.

With the advent of omics profiling explorative analysis of large sets of molecular features associated with disease development and progression became feasible. Generic analysis of omics datasets focuses on identification of individual molecular features exhibiting significant differences in abundance e.g. in case-control studies [11]. More recent approaches have complemented statistics-driven approaches with biological background knowledge e.g. in the form of protein-protein interaction data [12] aiming at expanding from molecular feature association towards identification of molecular mechanistic context. These strategies allow building descriptive molecular models of disease pathophysiology on the level of molecular processes and pathways [13, 14]. Specifically in oncology research various types of biological network models have been used to model pathophysiology, but also to identify and prioritize drug targets and molecular markers [15]. As example, association of differentially expressed genes in a set of human cancer cell lines resistant to methotrexate have been analyzed on a biological network level by Selga and colleagues, allowing identification of resistance-associated key proteins [16]. In a similar way network-based models approximating drug mechanism of action at the interface of disease molecular mechanisms have been introduced [17, 18]. In contrast to targeting individual molecular features involved in development of drug resistance such network-based approaches promise identification of more generic, underlying signaling components, in consequence improving coverage of individual resistance effector mechanisms.

In this work we present a HGSOC molecular process model resting on an interaction network of molecular features associated with platinum-based drug resistance as identified in transcriptomics studies, further complemented with protein coding genes mined from scientific publications. We use this model representation to identify core molecular processes afflicted with drug resistance specifically triggered by a combination therapy of platinum-based drugs and taxanes. With such drug resistance molecular processes as basis, screening for drug targets, drug mechanism of action interference and predictive biomarker candidates becomes feasible.

## Methods

**Fig. 1:**
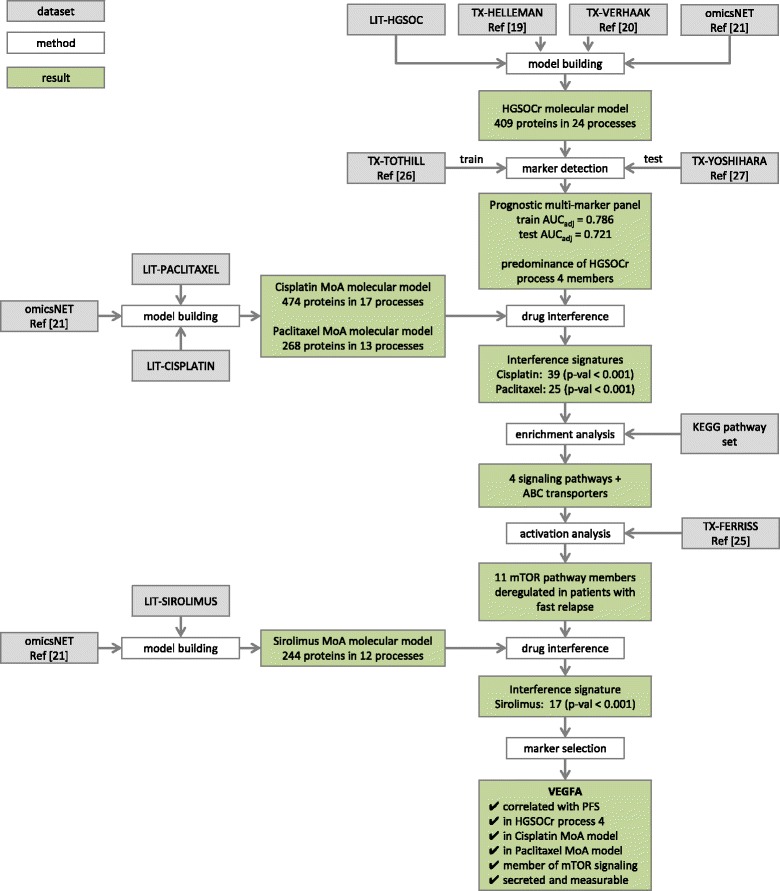
Data analysis workflow. Schematic representation of the data analysis workflow with used datasets, methods, and results indicated by grey, white, and green boxes respectively

A data analysis overview is depicted in Fig. 1 with individual analysis steps and results described in the following sections.

### Molecular data sources characterizing drug resistant HGSOC

Protein coding genes associated with drug resistant high-grade serous ovarian cancer were retrieved by querying the scientific literature, further extended by including results from transcriptomics studies. For mining scientific literature the following NCBI PubMed query was executed: *(“Ovarian Neoplasms/genetics” [majr]) OR (“Ovarian Neoplasms/pathology” [majr]) OR (“Ovarian Neoplasms/physiopathology” [majr]) OR (“Ovarian Neoplasms/enzymology” [majr]) OR (“Ovarian Neoplasms/chemistry” [majr]) OR (“Ovarian Neoplasms/metabolism” [majr]) AND (“recurrence”[mesh] OR “prognosis”[mesh] OR “drug resistance, neoplasm” [mh]) AND “humans” [mh]*. 5663 publications were identified and used to retrieve gene annotation provided by NCBI gene2pubmed, resulting in 777 unique human protein coding genes (LIT-HGSOC dataset).

This set of literature-based molecular features was complemented by protein coding genes derived from two transcriptomics studies in focus of disease relapse under platinum-based drug therapy. A first set of 432 molecular features was extracted from a study performed by Helleman et al., presenting a meta-analysis of nine individual transcriptomics studies, all focusing on HGSOC relapse in the context of platinum-based drug therapy (TX-HELLEMAN dataset) [19]. A second set of 100 molecular features was extracted from the cancer genome atlas providing a study on HGSOC published by Verhaak et al. [20] also focusing on cancer relapse under platinum-based drug therapy (TX-VERHAAK dataset). An overview of used datasets within this study is available in Table 1.

**Table 1:** Listing of used transcriptomics and literature mining datasets

Dataset acronym	Dataset description	Dataset use	Ref
LIT-HGSOC	Set of molecular features linked to HGSOC via literature mining.	Input for generating the HGSOCr molecular model	–
TX-HELLEMAN	Meta-analysis of nine transcriptomics studies reporting differentially regulated genes associated with ovarian cancer relapse.	Input for generating the HGSOCr molecular model	[19]
TX-VERHAAK	Transcriptomics dataset from The Cancer Genome Atlas reporting on differentially expressed genes linked with ovarian cancer disease prognosis.	Input for generating the HGSOCr molecular model	[20]
TX-FERRISS	Transcriptomics study on ovarian cancer patients to identify predictors of platinum resistance.	Input for evaluating the status of mTOR signaling pathway members	[25]
TX-TOTHILL	Transcriptomics study involving more than 200 ovarian cancer patients in order to identify molecular signature for subtyping ovarian cancer.	Training set for deriving the prognostic transcript panel	[26]
TX-YOSHIHARA	Transcriptomics study to identify survival signatures in serous ovarian cancer patients.	Test set for validating the prognostic transcript panel	[27]
LIT-CISPLATIN	Set of molecular features linked to cisplatin via literature mining.	Input for generating the cisplatin MoA molecular model	–
LIT-PACLITAXEL	Set of molecular features linked to paclitaxel via literature mining.	Input for generating the paclitaxel MoA molecular model	–
LIT-SIROLIMUS	Set of molecular features linked to sirolimus via literature mining.	Input for generating the sirolimus MoA molecular model	–

Overview and short description of datasets used for the integrated analysis in the present study. The specific use of the dataset in the integrated analysis is given along with the link to original publications for transcriptomics datasets

Finally, the three molecular feature sets LIT-HGSOC, TX-HELLEMAN, and TX-VERHAAK were combined into a set of 1,242 unique protein coding genes.

### Deriving a high-grade serous ovarian cancer resistance (HGSOCr) molecular model

Consolidated molecular features associated with drug resistant HGSOC were mapped on a hybrid interaction network including protein-protein interaction data from IntAct, BioGrid, and Reactome together with computationally inferred relations [21]. 1202 out of 1242 molecular features of the resistance molecular feature set could be assigned as nodes on the given network holding in total 15,905 protein coding genes. Subsequently, the HGSOCr specific induced subgraph was extracted, including all molecular features from the signature also holding an interaction to at least one other feature of the resistance molecular feature set. The induced subgraph, consisting of 1,062 protein coding genes, was forwarded to the Molecular Complex Detection algorithm for identifying clusters of nodes, in the following denoted as molecular processes [22, 23]. Including network interaction data between nodes across molecular processes forms a molecular model representation of drug resistant high grade serous ovarian cancer. This molecular model representation is composed of 24 individual molecular processes, each holding a set of highly interconnected proteins, embedding in total 409 proteins.

### Drug mechanism of action (MoA) molecular model computation and interference analysis with the HGSOCr molecular model

Cisplatin and paclitaxel are selected as representative compounds for platinum-based and taxane-based therapies, respectively. Drug mechanism of action molecular models were generated for these two compounds following the procedure applied for deriving the HGSOCr molecular model. Molecular feature sets characterizing drug effect were retrieved via gene2pubmed feature assignments to publications identified with the PubMed query *Cisplatin[mh]* (LIT-CISPLATIN dataset) and *Paclitaxel[mh]* (LIT-PACLITAXEL dataset), respectively. An additional drug MoA molecular model was derived for the mTOR inhibitor sirolimus applying the PubMed query *Sirolimus[mh]* (LIT-SIROLIMUS dataset).

Interference of a drug MoA molecular model and the HGSOCr molecular model is determined as number of molecular features being part of the respective drug MoA molecular model as well as being part of the HGSOCr molecular model.

### Pathway enrichment, activation status analysis and synthetic lethal interactions

Molecular pathway enrichment analysis using the Database for Annotation, Visualization and Integrated Discovery (DAVID) tool [24] was conducted for selected processes of the HGSOCr molecular model. The KEGG set of molecular pathways was used as underlying pathway resource, *p*-values were adjusted for multiple testing using the Benjamini-Hochberg correction method.

The transcriptomics dataset from Ferriss et al. [25] was used for evaluating the status of molecular pathways identified in enrichment analysis. The expression profiles were retrieved from the Gene Expression Omnibus (GEO) (GSE30161) and processed using the affy R package applying robust multiarray average (RMA) normalization (TX-FERRISS). Only patients with serous ovarian cancer were included in the calculations. Correlations in gene expression of pathway members to progression free survival were calculated in order to verify pathway relevance in drug resistance.

Synthetic lethal interactions of protein coding genes embedded in drug mechanism of action molecular models were retrieved from BioGRID. Interactions with experimental evidence tags “Synthetic Lethality” or “Negative Genetic” for the organisms Homo sapiens, Saccharomyces cerevisiae, Mus musculus, Gallus gallus, Caenorhabditis elegans, and Drosophila melanogaster were included. Orthology mapping from non-human model organisms to the corresponding human genes were based on orthology information as provided by Ensembl.

### Prognostic biomarkers included in the HGSOCr molecular model feature set

Two transcriptomics datasets, TX-TOTHILL and TX-YOSHIHARA, not included in deriving the HGSOCr molecular model were used in order to evaluate the prognostic potential (time to relapse) of molecular features embedded in the HGSOCr molecular model. Raw transcriptomics data files were retrieved from GEO for the studies of Tothill et al. (GSE9899, TX-TOTHILL dataset) [26] and Yoshihara et al. (GSE17260, TX-YOSHIHARA dataset) [27] together with data on time of progression free survival (PFS) as provided. Both studies focused on patients undergoing standard chemotherapy using platinum-based drugs in combination with taxanes. Pearson correlation coefficients of candidate biomarker expression levels and PFS given months were computed. Additionally, dichotomization was performed for allowing computation of area under the curve (AUC) values. For this, patients with PFS of less than 12 months were classified as the drug resistance cohort. The platinum based first line therapy takes 6 months and relapse within 6 months after the end of treatment is referred to as therapy resistance (12 months in total). Patients with PFS of more than 22 months were considered sensitive to chemotherapy. We focused on these two extremes thus excluding patient only partially responding to therapy in order to get a clearer picture on deregulated processes and markers. For the TX-TOTHILL dataset only patients with serous ovarian cancer treated with a platinum based therapy were included. 82 of the 226 patients had PFS of less than 12 months and 63 of the 226 patients had PFS of more than 22 months. The TX-YOSHIHARA dataset consists of 110 patients, all of being of type serous ovarian cancer and receiving platinum based therapy. 29 patients had PFS of less than 12 months and 45 had PFS of more than 22 months.

For retrieving expression profiles of resistant and sensitive patient cohorts the Affymetrix microarray data from TX-TOTHILL were processed using the affy R package applying RMA normalization.

For the Agilent-based data set TX-YOSHIHARA the R limma package was used for data preparation with the normexp background correction method and quantile normalization, averaging duplicate features after normalization.

The dataset TX-TOTHILL was used as training dataset in order to delineate a biomarker panel for assessing outcome on the level of progression free survival categorization. The dataset TX-YOSHIHARA was successively used as test dataset for the transcript feature panel delineated from the TX-TOTHILL dataset. Bootstrapping (200 runs) of least absolute shrinkage and selection operator (LASSO) logistic models was performed to calculate feature selection frequencies thus estimating individual feature relevance. The tuning parameter λ was selected in order to minimize cross-validation deviance. To assess the performance of the transcript feature panel an optimism adjusted area under the curve (AUC_adj_) value for a logistic model only using features from the transcript feature panel as explanatory variables was calculated for each of the two datasets with AUC_adj_ = AUC_obs_ – (AUC_boot_ – AUC_test_), where AUC_obs_ is the training AUC achieved on the entire dataset, AUC_boot_ is the training AUC achieved on the bootstrapped data set and AUC_test_ is the AUC of a predictor trained on the bootstrapped data set tested on the entire data set. All calculations were done in R using the packages GLMNET and ROC.

For annotation of biomarker candidates regarding evidence for being on a protein level detectable in circulation the following criteria were applied: (i) being identified as secreted or in the extracellular space based on UniProt subcellular localization annotation, (ii) being reported as measurable in blood based on NCBI pubmed MeSH annotation of any disease term with the MeSH qualifier *blood*, or (iii) being reported as biomarker in ovarian cancer based on publications annotated with the MeSH terms *(ovarian neoplasms[majr] AND (biological markers[mh] OR tumor markers, biological[mh]))*.

## Results

### Molecular representation of HGSOC resistance

The scientific literature search provides 777 molecular features associated with HGSOC resistance (LIT-HGSOC). Together with 432 features from the transcriptomics meta-study TX-HELLEMAN and 100 features as reported by Verhaak et al. (TX-VERHAAK), a unique set of 1242 molecular features is identified as associated with drug resistant HGSOC. Apparent is the minor overlap of the individual data sets, a frequent finding in data source assembly across source types [28], but also in comparative analysis of signatures generated in the same omics category as transcriptomics [29]. Data integration on interaction networks allows identifying a connected core feature set, which based on adding interactions includes a biological constraint on top of varying evidence of the features identified in individual experimental settings [30].

**Fig. 2:**
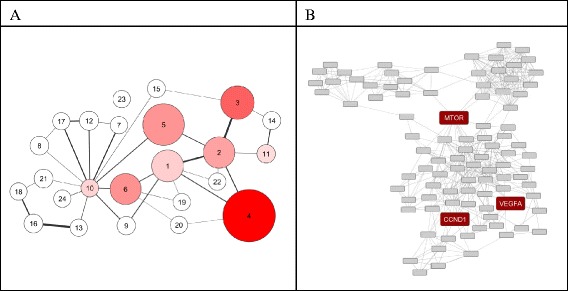
HGSOCr molecular model. **a** Each node represents a molecular process, the node diameter scales with the number of protein coding genes included. Edges between molecular processes indicate a significant number of protein-protein interactions between protein coding genes across molecular processes. Color-coding scales with the sum of individual biomarker frequencies in LASSO selection based on bootstrap runs of the transcript feature set classifier for explaining variance in PFS. **b** Subgraph representation of molecular process 4. Each node codes for a protein coding gene, edges represent interactions according to the underlying interaction network. Genes of specific relevance are highlighted in red (VEGFA, mTOR, CCND1)

Mapping the HGSOCr feature set on the selected hybrid interaction network results in an induced subgraph holding 1062 protein nodes. This subgraph resembles one giant component with a path from each protein coding gene (network node) to all other gene nodes. Applying a segmentation algorithm for identifying densely connected gene sets provides 24 molecular process segments holding in total 409 molecular features, with molecular process size ranging from 3 to 95 nodes (Fig. 2a).

Apparent is a significant reduction of the number of protein coding genes being included in the model representation, reflecting the interaction-based filtering of the initial feature set on the network level. Regarding relative contributions from the initial data sources 304 features in the molecular model link back to scientific literature, complemented by 138 features stemming from transcriptomics studies. Assuming improved evidence for representation of relevant mechanistic context on such molecular process level this molecular model representation and embedded protein coding genes are used in further analysis.

### A transcript feature set for tumor relapse prognosis

Forwarding the 409 protein coding genes of the molecular model to a LASSO logistic model for class prediction of sensitive and resistant samples according to profiling data of TX-TOTHILL (Table 2) provides a panel of 15 transcripts. The classifier achieves an optimism corrected AUC of 0.786 with the largest contribution of transcript features coming from molecular process 4 (Fig. 2), namely the nuclear receptor coactivator 3 (NCOA3), the transcription factor SPDEF, the transforming growth factor beta 2 (TGFB2) and the insulin-like growth factor 1 (IGF1). Next to representatives from molecular process 4 further transcripts included in the panel are the ephrin receptor 4 (EPHB4) from molecular process 1, the homeodomain interacting protein kinase 2 (HIPK2) and the forkhead box J1 (FOXJ1) from molecular process 2, the transcription factor TCF7L2, the phosphatase and tensin homolog (PTEN) and the homeobox 5 (HOXA5) from molecular process 3, a DNA replication licensing factor (MCM4) from molecular process 5, and the prostaglandin-endoperoxide synthase 2 (PTGS2) and the cyclin-dependent kinase inhibitor 1C (CDKN1C) from molecular process 6, complemented by the ELAV like RNA binding protein 1 (ELAVL1) and the chemokine (C-X-C motif) ligand 12 (CXCL12) from molecular processes 10 and 11, respectively.

**Table 2:** Clinical characteristics of samples and classifier performance

	TX-TOTHILL	TX-YOSHIHARA
	Training set	Test set
number of patients	145	71
no relapse > 22 months (sensitive)	63	42
relapse < 12 months (resistant)	82	29
average PFS	21.69 +/− 21.47	25.86 +/− 21.68
PFS, sensitive group	39.33 +/− 22.40	40.21 +/− 16.72
PFS, resistant group	8.13 +/− 2.77	5.07 +/− 3.09
FIGO stage		
early stage (I-IIA)	15	0
advanced stage (IIB-IV)	130	71
Chemotherapy		
platinum-based drug	29	0
platinum-based drug and taxanes	116	71
array platform	Affymetrix Human Genome U133 Plus 2.0	Agilent Whole Human Genome Microarray 4x44K
optimism corrected AUC	0.786	0.721

Clinical characteristics of the patient samples used in transcriptomics profiling and performance of the prognostic transcript panel classifier in identifying sensitive and resistant specimens

This classifier holding 15 transcripts was evaluated in the independent transcriptomics dataset TX-YOSHIHARA, reaching an optimism corrected AUC value of 0.721.

Evaluating selection probabilities of biomarkers according to the LASSO procedure identified biomarker candidates associated with molecular process 4 as most relevant in both, training and test datasets for linking with relapse.

### Cisplatin and paclitaxel MoA molecular models and interference with the HGSOCr molecular process 4

According to data inclusion criteria the HGSOCr molecular model rests explicitly on molecular features identified in the context of resistance to standard chemotherapy utilizing platinum-based drugs together with taxanes. As molecular process 4 holds key biomarkers for adding to an explanation of variance in PFS, the entire molecular process warrants further analysis in the light of drug mechanism of action of cisplatin and paclitaxel.

**Fig. 3:**
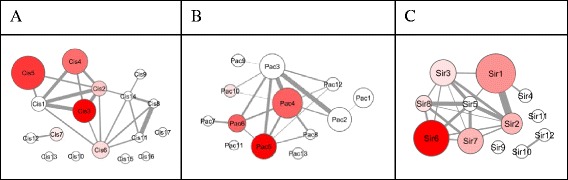
Drug mechanism of action molecular models. Molecular model representation of (**a**) cisplatin, (**b**) paclitaxel, and (**c**) sirolimus mechanism of action. Each node represents a molecular process, the node diameter scales with the number of protein coding genes included. Edges between molecular processes indicate a significant number of protein-protein interactions between protein coding genes across molecular processes. Color-coding scales with number of overlapping nodes with respect to nodes embedded in molecular process 4 of the HGSOCr molecular model

The cisplatin MoA molecular model holds 474 molecular features organized in 17 molecular processes, respective numbers for the paclitaxel MoA molecular model are 268 molecular features in 13 molecular processes (Fig. 3a, b).

39 nodes of the cisplatin MoA molecular model are also part of the HGSOCr molecular process 4, and 25 nodes are shared by the paclitaxel MoA molecular model and the HGSOCr molecular process 4. In total 43 of the 95 HGSOCr molecular process 4 members are either in the cisplatin and/or the paclitaxel MoA molecular model, resulting with respect to model sizes in a highly significant overlap according to a Chi2 test. Apparently, drug mechanism of action and association with relapse are linked in molecular process 4 of the HGSOCr molecular model.

The overlapping features resemble several genes reported to be involved in drug resistance including ABC transporters, the serine/threonine kinase mTOR, the apoptosis inhibitor BCL2, the vascular endothelial growth factor A (VEGFA), members of the STAT family, IL6 and tumor necrosis factor alpha (TNF).

Computing KEGG pathway enrichment for the set of 95 protein coding genes in HGSOCr process 4 results in eleven significantly enriched pathways as listed in Table 3. Five of these KEGG pathways are also significantly enriched on the basis of the 43 protein coding genes showing overlap with the two drug mechanism of action molecular models of cisplatin and paclitaxel.

**Table 3:** KEGG pathway enrichment

Pathway	# Genes	Estimate	*p*-value
**hsa04350:TGF-beta signaling pathway**	**11**	**11.58**	**1.00E-05**
hsa03010:Ribosome	11	11.58	1.00E-05
**hsa04150:mTOR signaling pathway**	**8**	**8.42**	**1.04E-04**
**hsa02010:ABC transporters**	**7**	**7.37**	**3.36E-04**
**hsa04630:Jak-STAT signaling pathway**	**9**	**9.47**	**0.00901**
hsa04620:Toll-like receptor signaling pathway	7	7.37	0.01620
hsa04010:MAPK signaling pathway	11	11.58	0.01982
hsa04914:Progesterone-mediated oocyte maturation	6	6.32	0.03358
hsa04012:ErbB signaling pathway	6	6.32	0.03330
**hsa04722:Neurotrophin signaling pathway**	**7**	**7.37**	**0.03265**
hsa04060:Cytokine-cytokine receptor interaction	10	10.53	0.04148

Pathways according to KEGG computed as significantly enriched in molecular process 4 of the HGSOCr molecular model. Pathways given in bold are further identified as enriched in the drug mechanism of action model overlap feature set of cisplatin and paclitaxel matching with molecular process 4 members of the HGSOCr molecular model

Four signaling pathways are among the five pathways, namely TGF-beta, mTOR, Jak-STAT, and Neurotrophin signaling. With two features of the prognosis classifier attributable to mTOR signaling (IGF1 and PTEN) specific focus is on the mTOR pathway as link with drug resistance and relapse.

### Regulation of the mTOR pathway and synthetic lethal interactions

For evaluating relevance of the mTOR pathway with respect to relapse, correlation with expression values of mTOR pathway members in the TX-FERRISS data set is determined. Eleven mTOR pathway members show correlation coefficients regarding PFS of > = 0.4 or < = −0.4, with expression of seven genes being negatively correlated and four being positively correlated with PFS (Table 4). VEGFA shows the strongest correlation to PFS with a Pearson R of −0.57 thus indicating higher levels of VEGFA in patients with reduced PFS. Next to VEGFA, the hypoxia inducible factor 1 alpha is negatively correlated with PFS, being activated by mTOR and itself being an activator of VEGFA.

**Table 4:** Correlation of mTOR members to progression free survival

Affymetrix transcript ID (Affymetrix Human Genome U133 Plus 2.0)	Gene symbol	Pearson correlation coefficient, PFS
210512_s_at	VEGFA	−0.57
202887_s_at	DDIT4	−0.53
212688_at	PIK3CB	−0.51
220587_s_at	MLST8	0.49
41657_at	STK11	0.46
226312_at	RICTOR	−0.45
206598_at	INS	0.43
213404_s_at	RHEB	−0.42
200989_at	HIF1A	−0.42
212609_s_at	AKT3	−0.42
242674_at	EIF4E	0.41

Pearson R correlation (> = 0.4 and < = −0.4) of mTOR members regarding PFS. Provided are gene symbols, Affymetrix transcript IDs of the TX-FERRISS dataset, and Pearson correlation coefficients

Apparent involvement of the mTOR pathway in drug resistance provides a rationale for evaluating mTOR inhibitors, selecting sirolimus as example. According to molecular model interference of HGSOCr process unit 4 and a sirolimus MoA molecular model (Fig. 3c), highly significant overlap is seen. 17 nodes of the sirolimus MoA molecular model see matches in molecular process 4, next to covering key elements of the mTOR pathway as such also addressing ErbB- and neutrophin-signaling as well as ABC transporters.

Next to directly addressing the mTOR context via sirolimus in relation to cisplatin and paclitaxel mechanism of action, synthetic lethal interactions induced by such drug combination may add to efficacy in tackling resistance. Specifically, such effect may be targeted to drug resistant cells potentially limiting toxic effects of such triple drug combination in non-cancer cells.

According to molecular model interference cisplatin holds 39 nodes in the HGSOCr molecular process 4, the respective numbers for paclitaxel and sirolimus are 25 and 17. Screening for synthetic lethal candidate interactions among such molecular process 4 members identifies a coupling of sirolimus and paclitaxel, involving a protein kinase C (PRKCH) and the synthetic lethal partners cyclin D1 (CCND1) and a second protein kinase C (PRKCI).

### A predictive biomarker for patient stratification

Biomarkers in their definition allow probing the status of a specific molecular process, in the given focus ideally informing on the mTOR pathway specifically at the interface of platinum-based drug and taxane mechanism of action. VEGFA exhibits strongest correlation with PFS, is embedded in molecular process 4 of the HGSOCr molecular model, directly linked with the mTOR pathway, and embedded in both, the cisplatin as well as paclitaxel MoA molecular model. On top, the candidate is secreted and measurable in circulation, hence allowing minimally invasive evaluation in patient samples.

## Discussion

A major challenge in clinical management of ovarian cancer is development of resistance against first line therapy, in consequence leading to tumor relapse in the majority of cases. We in this work present a data integration workflow for identifying relevant molecular processes characterizing drug resistance in HGSOC with respect to first line chemotherapy, from there linking with alternative drug mechanism of action and candidate predictive biomarkers.

Utilizing a molecular network approach allows deriving a molecular model approximating key molecular context of the drug resistant phenotype, composed of 24 molecular processes in total embedding 409 protein coding genes. Relating molecular processes to PFS via a biomarker panel-based classifier gives rise to further analyze a specific molecular process holding in total 95 molecular features. Functional characterization of this gene set provides individual mediators of drug resistance as ABC transporters, but also specific signaling pathways, namely TGF-beta, mTOR, JAK-STAT and Neutrophin signaling. These pathways are of specific interest, being not only embedded in the HGSOCr molecular model but also in the molecular mechanism of action models of cisplatin and paclitaxel - against which resistance developed in the first place.

Up to now tackling individual resistance mediators as ABC transporters failed in clinical testing, e.g. seeing increased toxicity and unexpected alterations in the pharmacokinetics of antineoplastic drugs when administered together with ABC inhibitors [31]. These findings may point towards alternatively addressing signaling pathways for overcoming drug resistance. With respect to the specific signaling pathways identified in molecular process 4 of the HGSOCr molecular model, involvement in cancer progression and drug resistance is a common theme. TGF-beta signaling carries out a dual role during the progression of cancer. One of its key functions is to maintain homeostasis of several cell types like epithelial, endothelial, and hematopoietic cells, therefore acting as tumor suppressor in the early stages of cancer through the induction of cell cycle arrest and apoptosis [32]. Later in cancer development, due to oncogenic mutations in this pathway, TFG-beta becomes capable of promoting tumor growth and metastatic functions via epithelial to mesenchymal transition induction. This transition promotes cell migration and invasiveness, and is considered a key step in the acquisition of resistance to chemotherapeutic agents [33].

The JAK/STAT pathway is a crucial mediator of the cellular responses to cytokines and growth factors and involved in the regulation of cellular processes such as cell growth, differentiation, apoptosis, development, and immune response [34]. Not surprisingly it is found to be altered in several cancer types and involved in drug resistance and invasiveness [34]. STAT3, robustly activated by interleucin-6, is well studied for promoting tumor progression and poor prognosis [35].

The closely related family of neurotrophins is involved in the survival, development, and function of neurons. Through the tropomyosin-related kinase (Trk) family of tyrosine receptors (TrkA, TrkB, and TrkC) they modulate multiple signaling pathways through the activation of proteins like PI3K, Ras, MAPK or NF-kB, rendering neutrophins highly relevant in development of chemotherapy resistance [36]. Increased expression of Trks, especially TrkB, and correlation to poor prognosis is reported in a number of cancers including ovarian cancer [37].

Specific attention is with the mTOR pathway, found to be activated in about half of the high grade serous ovarian cancer patients [38]. The pathway holds a central role in conferring environmental signals to regulate growth and homeostasis. mTOR itself is part of two large protein complexes (mTORC1 and mTORC2). mTORC1 integrates input from growth factors, extra- and intracellular stress, oxygen and energy status and amino acid levels. Activation is mainly triggered via regulation of its inhibitors TSC1/2 [39]. Oncogenic mutations in this pathway are, in contrast to other ovarian cancer subtypes, rare in HGSOC. The constitutive activation in ovarian cancer is a result of amplification of pathway components like PI3K subunits (p110), AKT isoforms (AKT1, AKT2, or AKT3), or members of the mTOR complexes (RICTOR, RAPTOR). Other factors include deregulation of upstream receptor tyrosine kinases (ERBB3, ERBB2, IGF1R, EGFR), or the cross-talk with the Ras pathway through amplification of KRAS or MAPK [40]. The consequences of an upregulated PI3K/AKT/mTOR pathway are extended cell survival, further reported as being involved in drug resistance [41]. The levels of pPIK3CA and pAkt are correlated with a decreased survival in ovarian cancer [42]. Mutations in PIK3CA and loss of the tumor suppressor PTEN are shown to initiate ovarian tumorgenesis in mice, being reversible by PI3K/AKT/mTOR pathway inhibition [43].

Analysis of drug mechanism of action molecular models of cisplatin and paclitaxel on the background of molecular processes characterizing resistant HGSOC results in a number of genes associated with mTOR signaling including mTOR itself, but also IGF1, VEGFA, or MAPK1. Several studies support an increased activity of the PI3K/AKT/mTOR pathway upon platinum or paclitaxel based treatment. In in-vitro studies of ovarian cancer cells, cisplatin addition leads to AKT phosphorylation and AKT/mTOR pathway induction. Cisplatin resistant cells generated by sustained drug exposure result in elevated levels of AKT/mTOR pathway components [44]. The same is observed in colon and cervical cancer cells. Cisplatin administration leads to AKT1, mTOR, S6K, and 4E-BP1 phosphorylation and hence pathway activation [45, 46]. The clear cell carcinoma subtype, in contrast to serous adenocarcinomas, shows a response rate of only around 11 % to standard first line therapy indicating an intrinsic drug resistance. Interestingly in about 87 % of 52 tested clear cell carcinomas elevated mTOR activity is observed [47]. Inhibition of the mTOR pathway, in most cases through PI3K inhibitors, could sensitize the cells to cisplatin [46, 47].

Paclitaxel is found to activate the mTOR pathway in cervical cancer cell lines and its inhibition sensitizes the cells to treatment [48]. Also in an ovarian cancer cell line as well as in xenograft models impeded mTOR pathway activity increases the efficacy of paclitaxel [49].

A study addressing the synergistic effect of sirolimus with cisplatin, paclitaxel, gemcitabine, or etoposide in different ovarian cancer cell lines reveals a cell line dependent response, however, seeing agonistic as well as antagonistic effects [50]. These findings pinpoint two essential elements to be considered when targeting the mTOR pathway. First it is pivotal to utilize a suitable biomarker to identify effective activity of the pathway. As evidenced in different ovarian cancer cell lines and as noted before also in human tissue samples mTOR pathway activation is not an ubiquitous finding in ovarian cancer drug resistance, hence stratification via a biomarker is necessary. Second, target specificity of the drug needs consideration, as sirolimus for example only targets the mTORC1 but not the mTORC2 complex, the latter triggering a positive feedback on Akt [51].

Screening members of the mTOR pathway in a transcriptomics dataset for exhibiting correlation with progression free survival reveals several candidates. Activators of mTOR including PIK3CB, AKT3, or RHEB are upregulated, suppressors as STK11 are downregulated with respect to earlier relapse. RICTOR, a mTORC2 complex member, is also identified as upregulated, and reported as associated with cisplatin resistance through the inhibition of AKT degradation in ovarian cancer cells, and respective downregulation sensitized cells to cisplatin [52]. Additionally, several targets of mTOR are found to be negatively correlated with PFS including HIF1A and downstream VEGFA and DDIT4. Interestingly EIF4E, upregulated by mTOR, is found positively correlated with PFS.

VEGFA shows the strongest correlation with progression free survival and also its activator HIF1A exhibits a significant upregulation upon early relapse. Several studies reveal a connection between the HIF1A/VEGFA axis in drug resistance and poor prognosis in various cancer types including ovarian carcinoma [53, 54]. Tumor development usually involves a hypoxic state sensed by the mTOR pathway leading to an activation of the hypoxia-inducible factor 1 alpha (HIF1A) to facilitate angiogenesis upon VEGFA induction [55]. VEGFA abundance not only shows the strongest correlation with PFS in our analysis but also fulfils criteria to qualify as a biomarker, namely being secreted and detectable in blood. A very recent study in high grade serous ovarian carcinoma documented the correlation of VEGFA levels and poor prognosis, rendering VEGFA an ideal candidate to stratify patients prone to drug resistance upon mTOR pathway upregulation [56].

Several clinical trials have been performed in ovarian cancer patients targeting the mTOR pathway, including monotherapies as well as combination with standard first line therapy, however, resulting in limited benefit [40]. Data from a phase 2 trial of temsirolimus in persistent and recurrent epithelial ovarian and primary peritoneal malignancies showed only modest efficacy in unselected patients and the study authors concluded that only with the inclusion of patient stratification markers further studies are warranted [57].

In recent years inhibitors of PI3K/Akt and anti-angiogenics as e.g. bevacizumab addressing VEGF have received attention for allowing a more effective inhibition of signal transduction in the pathway [40, 58], further combined with mTOR inhibitors for achieving a comprehensive inactivation of the mTOR pathway [59]. These strategies aim at interfering with compensatory feedback loops, like the Akt activation via mTORC2 when mTORC1 is inhibited by rapalogs, or the activation of the insulin growth factor 1 receptor when its inhibitor mTOR is blocked [60]. Also the cross talk to other pathways like the RAS/MAPK cascade may counteract drug effect through Erk1/2 activation upon mTOR inhibition [61]. A further strategy follows dual mTORC1/mTORC2 inhibitors, with preclinical data demonstrating inhibition of ovarian cancer cell proliferation specifically when combined with paclitaxel [62]. Here synthetic lethal interactions may add to efficacy, including CCND1 as well as PRKCI, both associated with poor prognosis [63, 64]. Specifically cyclin D1 appears relevant, being proposed as selection marker for mTOR inhibitor treatment according to phase II study results [57].

## Conclusions

Integrating molecular data characterizing drug resistant HGSOC in a molecular process model identifies the mTOR pathway as relevant component mediating resistance in the context of cisplatin and paclitaxel drug mechanism of action, and proposes mTOR inhibitors as means for addressing resistant phenotypes. Clinical trials following such approach identified modest activity, clearly addressing the need for stratification markers. VEGFA as well as cyclin D1 result as candidate biomarkers for stratification, and combined with dual mTOR inhibitors warrant further experimental testing.

### Ethics approval and consent to participate

Not applicable.

### Consent for publication

Not applicable.

### Availability of data and material

Omics datasets used in this study are available in the Gene Expression Omnibus (http://www.ncbi.nlm.nih.gov/geo/) (GSE dataset numbers provided in the text) and at The Cancer Genome Atlas (http://cancergenome.nih.gov/). Generated molecular model files are available upon request from the authors.

## Abbreviations

AUC: Area under the Curve
DAVID: Database for Annotation, Visualization and Integrated Discovery
GEO: Gene Expression Omnibus
HGSOC: High Grade Serous Ovarian Cancer
HGSOCr: High Grade Serous Ovarian Cancer, drug resistant phenotype
LASSO: Least Absolute Shrinkage and Selection Operator
MeSH: Medical Subject Heading
MoA: Mechanism of Action
PFS: Progression Free Survival
RMA: Robust Multiarray Average
ROC: Receiver Operating Characteristic
